# Lack of Association between Inadequate Micronutrient Intake and Prognosis in Outpatients with Heart Failure

**DOI:** 10.3390/nu14040788

**Published:** 2022-02-13

**Authors:** Núbia Rafaella Soares Moreira Torres, Fernanda Lambert de Andrade Freire, Raquel Costa Silva Dantas-Komatsu, Eduardo Paixão da Silva, Salomão Israel Monteiro Lourenço Queiroz, Niethia Regina Dantas de Lira, Rosiane Viana Zuza Diniz, Severina Carla Vieira Cunha Lima, Lucia Fatima Campos Pedrosa, Márcia Marília Gomes Dantas Lopes, Karine Cavalcanti Maurício Sena-Evangelista

**Affiliations:** 1 Postgraduate Program in Nutrition, Center for Health Sciences, Federal University of Rio Grande do Norte, 3000, Senador Salgado Filho Avenue, Lagoa Nova, Natal 59078-970, Rio Grande do Norte, Brazil; rafaellamoreira@hotmail.com (N.R.S.M.T.); nandalambert@gmail.com (F.L.d.A.F.); severina.lima@ufrn.br (S.C.V.C.L.); lucia.pedrosa@ufrn.br (L.F.C.P.); 2 Postgraduate Program in Health Sciences, Center for Health Sciences, Federal University of Rio Grande do Norte, 620, Nilo Peçanha Avenue, Petrópolis, Natal 59012-300, Rio Grande do Norte, Brazil; raquelcsdantas@gmail.com; 3 Multiprofessional Residency in Health—Cardiology, Onofre Lopes University Hospital, Federal University of Rio Grande do Norte, 620, Nilo Peçanha Avenue, Petrópolis, Natal 59012-300, Rio Grande do Norte, Brazil; edu-paixao-96@hotmail.com (E.P.d.S.); niethialira@gmail.com (N.R.D.d.L.); marilia.lopes@ufrn.br (M.M.G.D.L.); 4 Postgraduate Program in Public Health, Health Sciences Center, Federal University of Rio Grande do Norte, 3000, Senador Salgado Filho Avenue, Lagoa Nova, Natal 59078-970, Rio Grande do Norte, Brazil; salomaoisrael10@gmail.com; 5 Brazilian Hospital Services Company, Onofre Lopes University Hospital, Federal University of Rio Grande do Norte, 620, Nilo Peçanha Avenue, Petrópolis, Natal 59012-300, Rio Grande do Norte, Brazil; 6 Department of Clinical Medicine, Center for Health Sciences, Federal University of Rio Grande do Norte, 620, Nilo Peçanha Avenue, Petrópolis, Natal 59012-300, Rio Grande do Norte, Brazil; rosianevzdiniz@gmail.com; 7 Department of Nutrition, Center for Health Sciences, Federal University of Rio Grande do Norte, 3000, Senador Salgado Filho Avenue, Lagoa Nova, Natal 59078-970, Rio Grande do Norte, Brazil

**Keywords:** heart failure, food intake, micronutrients, hospitalization, mortality

## Abstract

Inadequate nutrient intake can lead to worse outcomes in patients with heart failure (HF). This prospective cohort study aimed to assess the prevalence of inadequate micronutrient intake and their association with prognosis in 121 adult and elderly outpatients with HF. Habitual micronutrient intake was evaluated using 24-h dietary recalls (minimum 2 and maximum 6). Participants were grouped into moderate (*n* = 67) and high (*n* = 54) micronutrient deficiency groups, according to the individual assessment of each micronutrient intake. Patients’ sociodemographic, clinical, and anthropometric data and clinical outcomes (hospitalization and mortality) within 24 months were collected. Overall and event-free survival rates were calculated using Kaplan–Meier estimates, and curves were compared using the log-rank test. The death risk rate (hazard ratio (HR)) was calculated using Cox’s univariate model. The rate of inadequate intake was 100% for vitamins B1 and D and above 80% for vitamins B2, B9, and E, calcium, magnesium, and copper. No differences in overall survival and event-free survival were observed between groups of HF outpatients with moderate and high micronutrient deficiencies (HR = 0.94 (CI = 0.36–2.48), *p* = 0.91, and HR = 1.63 (CI = 0.68–3.92), *p* = 0.26, respectively), as well as when the inadequacy of each micronutrient intake was evaluated alone (all *p* > 0.05). In conclusion, a high prevalence of inadequate micronutrient intake was observed in outpatients with HF. Inadequate micronutrient intake was not associated with hospitalization and mortality in this group of patients.

## 1. Introduction

Heart failure (HF) is a clinical syndrome characterized by cardiac abnormalities and impaired blood supply for tissue demands. HF is an important public health problem that affects more than 23 million people worldwide, with a high rate of morbidity and mortality [1]. The leading causes of HF are high blood pressure, diabetes mellitus, metabolic syndrome, and coronary artery disease [2].

Malnutrition is a common problem in patients with advanced stages of HF [3]. Conversely, being overweight is also a typical nutritional disorder in this population [4]. However, there is a clear risk of coexistence of micronutrient deficiency along with overweight and obesity [5]. It was observed that HF patients with a greater number of micronutrient inadequacies had worse survival. Thus, adequate nutritional intake is considered to be an essential component of management and prevention of unfavorable clinical outcomes [6].

There are several psychological, social, and HF-related factors that can affect food consumption, e.g., decreased hunger sensations, diet restrictions, fatigue, shortness of breath, nausea, and intestinal edema, resulting in poor nutrient absorption [7,8]. Therefore, patients with HF commonly have an inadequate intake of several micronutrients, such as vitamins A, B1, B2, and D; calcium; potassium; magnesium; zinc; copper; selenium; and iodine [9,10,11]. These nutrients play a fundamental role in several mechanisms for the maintenance of cardiac activity, such as acting on the systolic function of the left ventricle [12], antioxidant and anti-inflammatory actions, modulation of the autonomic adrenergic response, endothelial function, carbohydrate metabolism [11], anti-hypertrophic effects, regulation of the extracellular matrix, regulation of metalloprotein expression, and regulation of calcium flow and cardiac contractility [12]. Despite the fundamental role of micronutrients in the context of HF, there is no specific reference intake for this group of individuals or dietary guidelines for patients with HF [13,14]. On the other hand, some authors have suggested that micronutrient supplementation could be beneficial for myocardial function, delaying the occurrence of non-reversible adverse events, in addition to bringing benefits to nutritional status [15]. However, studies are inconclusive about supplementation protocols [3].

Gaps regarding aspects that interfere with the occurrence of adverse events in HF still exist, which can increase or reduce the survival time free from cardiac events. In addition, food and dietary intake have been investigated as factors associated with adverse events in patients with HF [16]. However, there is a scarcity of studies addressing the real scenario of micronutrient intake inadequacy in individuals with HF and the impact on clinical outcomes. The findings from this type of study could provide relevant insights for future intervention strategies. Thus, this study aimed to assess the prevalence of inadequate micronutrient intake and its association with prognosis in adult and elderly outpatients with HF.

## 2. Materials and Methods

### 2.1. Study Population and Design

This prospective cohort study was performed at the Heart Failure Clinic of Onofre Lopes University Hospital of the Federal University of Rio Grande do Norte, northeast Brazil. The study sample included 121 patients diagnosed with HF according to the Boston point system and Framingham criteria [1], confirmed by Doppler echocardiography. Adolescents, pregnant females, and patients with HF decompensation, using enteral and/or parenteral nutritional therapy, with chronic kidney disease undergoing hemodialysis or estimated glomerular filtration rate (eGFR) of <15 mL/min/1.73 m^2^, undergoing cancer or chemotherapy, with liver disease and/or thyroid disorders, with cognitive impairment, and who had undergone bariatric surgery were not included.

Participants were selected using non-probability sampling. All 171 patients who received treatment between April 2017 and March 2020 were considered eligible. Of these, 128 patients met the inclusion criteria and were invited to participate in the study, 4 of whom refused to participate. Of the 124 patients who agreed to participate in the study, 1 dropped out and 2 had only one 24-h dietary recall, resulting in a sample of 121 patients who were followed up for a period of 24 months after the first approach.

Initially, the participants sociodemographic information was obtained, and the first 24-h dietary recall was applied. In the second stage, after 30–45 days from the first stage, a second 24-h dietary recall was performed. Weight and height were measured to calculate body mass index (BMI), and blood sample was collected to analyze creatinine concentrations. Creatinine levels were measured using the kinetic method. eGFR was calculated using the Chronic Kidney Disease Epidemiology Collaboration equation, as defined by the International Society of Nephrology [17]. Subsequently, 24-h dietary recalls are applied.

Information on HF etiology, New York Heart Association (NYHA) functional classification, heart rate, and associated comorbidities were obtained from the patients’ electronic health records, whereas, left ventricular ejection fraction (LVEF) and type of HF (<40%, HF with reduced ejection fraction (HFrEF); 40–49%, HF with mid-range ejection fraction (HFmEF); and ≥50%, HF with preserved ejection fraction (HFpEF)) [18] were determined from the Doppler echocardiography report. Clinical evolution, hospitalizations, and mortality were collected by consulting electronic medical records or by telephone.

This study was approved by the Research Ethics Committee of Onofre Lopes University Hospital (CAAE 59827516.2.0000.5292, no. 3.769.093). All participants provided written informed consent.

### 2.2. Assessment of Dietary Intake

Data on habitual food intake were obtained using three 24-h dietary recalls performed according to Thompson and Byers [19]. An average of three 24-h dietary recalls (minimum of 2 and maximum of six 24-h dietary recalls) were obtained for all participants.

The quantity of each food item and drink was converted into grams or milliliters using a measurement chart for food consumed in Brazil. The foods were converted into energy and nutrients using the Virtual Nutri Plus^®^ 2.0 (São Paulo, Brazil). New preparations and foods were added to the software database as necessary, along with their nutrient composition obtained from the Brazilian food composition tables and the United States Department of Agriculture database, as appropriate [20,21,22,23]. Nutritional information from industrial food labels was also included in the software database.

### 2.3. Prevalence of Inadequate Micronutrient Intake

The nutritional information from 24-h dietary recalls was inserted in the multiple source method (https://msm.dife.de/ accessed 15 May 2021) [24] to calculate the usual dietary intake and then construct the population distribution based on these data. Nutrient intakes were then adjusted for total energy intake using the method proposed by Willet and Stamper [25].

The prevalence of the inadequate intake of vitamins A, B1, B2, B3, B6, B9, B12, C, D, and E; calcium; copper; iron; phosphorus; iodine; magnesium; selenium; sodium; and zinc was estimated using the estimated average requirement (EAR) cut-point method [24,25]. The prevalence of inadequate iron intake was calculated using a manually determined probabilistic approach. It was not possible to calculate the prevalence of potassium, manganese, and vitamin B5 as the EAR, because the values were not established. In this case, the “adequate intake” (AI) value was used as a cutoff point, and the frequency of inadequacy was calculated [26,27,28,29]. The recommendations of the Brazilian HF guidelines were used to assess sodium intake [1].

### 2.4. Clinical Outcome Assessment

From April to June 2020, the patients’ electronic health records were checked to collect clinical outcome information such as hospital admissions for clinical HF decompensation or cardiac complications (cardiac arrhythmias, coronary artery disease requiring cinecoronariography, valve replacement indications, or hemochromatosis) and deaths from all causes occurring between 2017 and 2020. A follow-up period of 24 months was considered for all patients. Information was also collected using a mobile phone.

### 2.5. Statistical Analysis

The sample calculation was performed assuming an alpha error of 5% and a beta error of 20%, obtaining a total of 92 people per group, to detect the difference in event-free survival of patients in the moderate and high micronutrient deficiency groups.

The nutrient intake of each patient was compared with the EAR value of the respective nutrient, considering the sex and age groups, and analyzed according to the classification “adequate” or “inadequate.” Patients were divided into two groups: moderate micronutrient deficiency group (up to 14 nutrients with inadequate intake) and high micronutrient deficiency group (>14 nutrients with inadequate intake). To define this cutoff point, the 50th percentile of the number of nutrients with inadequate intake in the sample was used. Subsequently, associations with health outcomes were made, considering these groups and the classification of micronutrient consumption individually. Continuous variables are expressed as means (standard deviation) for data with a normal distribution and medians (1st–3rd quartile) for skewed data. Categorical variables are expressed as absolute frequencies (percentage frequency) and 95% confidence intervals. For quantitative variables, the normality of distribution was verified using the Kolmogorov–Smirnov test for the application of parametric and nonparametric tests. For quantitative variables, the *t*-test was performed, and for categorical variables, the chi-square or Fisher’s exact test, when appropriate.

The overall survival time was calculated considering the interval between the date of the first outpatient visit and the date of death or the end of follow up. The event-free survival time was calculated by considering the individual’s initial assessment of the clinical outcome (hospitalizations or death). Event-free survival is defined as the time elapsed until the first adverse cardiac event and is used to assess the effectiveness of interventions in patients with HF [30]. The maximum follow up was 24 months, and patients who remained alive after this period were censored. The date of censorship for patients who did not die was considered as the end of the follow-up period. Survival analysis was performed using the Kaplan–Meier method and log-rank test to assess the statistical significance of the difference between the survival curves obtained. The overall survival and event-free survival curves for the 24-month follow up were constructed for the studied variables. The risk of death rates (hazard ratio) was calculated using univariate Cox models. The variables considered for univariate analysis are vitamin A, B2, B3, B6, B9, B12, vitamin C, E, calcium, iron, phosphorus, iodine, magnesium, manganese, potassium, selenium, sodium, zinc, which were categorized into adequate and inadequate. Additionally, micronutrient deficiency groups (moderate and high) were considered for analysis. Subsequently, Pearson’s chi-square and Fisher’s exact tests were performed to identify the associations and differences between variables. The analyses were performed using SPSS version 25.0 (Statistical Package for the Social Sciences, Chicago, IL, USA).

## 3. Results

In the overall group, patients were predominantly male; 48.2% were overweight/obese (average BMI = 30.80 kg/m^2^ (SD = 3.85)); 40.5% had HF of ischemic etiology, 59.3% had HFrEF; and 88.6% patients had NYHA class I/II. In the moderate micronutrient deficiency group, we observed a higher frequency of females (*p* = 0.001), HF of ischemic etiology (*p* = 0.003), diabetes mellitus (*p* = 0.01), and use of oral hypoglycemic agents (*p* = 0.02). There was a higher percentage of men and ex-drinkers in the high micronutrient deficiency group (all *p* < 0.05). Over the median follow up of 24 months, 17 patients died, and 8 patients were hospitalized (Table 1).

All patients had inadequate intake of vitamins B1 and D. Moreover, the proportion of 100% prevalence of inadequate vitamin B12 intake in females and inadequate calcium intake in males aged >51 years and females aged between 19 and 50 years was identified. Females aged >31 years also had 100% prevalence of inadequate magnesium intake. The prevalence of inadequate micronutrient intake >80% was found for vitamins B2, B9, and E and for calcium, copper, and magnesium. A higher prevalence of inadequate intake of zinc (82.4%) was found in males than in females (44.0%). The mean intake of potassium, vitamin B5, and manganese was lower than that of the AI. Further, 100% of females and 96.3% of males had sodium intake in accordance with the recommended Brazilian HF guidelines (Table 2).

**Figure 1:**
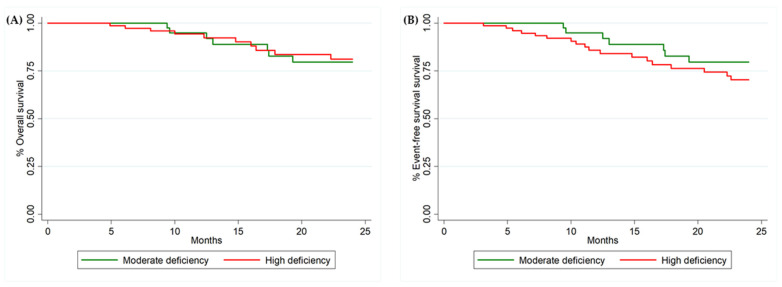
Kaplan–Meier curves for patients with HF considering (**A**) the overall survival and micronutrient deficiency groups and (**B**) event-free survival and micronutrient deficiency groups.

Overall survival was longer in patients with inadequate potassium intake than in those with adequate potassium intake (HR = 8.82 (CI 1.08–72.14), *p* = 0.01). However, only one individual had adequate intake of this micronutrient, who died within 24 months. There were no associations between the micronutrient deficiency categories, when assessed individually, with overall survival and event-free survival (all *p* > 0.05) (Table 3). There were also no significant differences in overall survival (HR = 0.94 (CI 0.36–2.48), *p* = 0.91) and event-free survival (1.63 (CI 0.68–3.92), *p* = 0.26) between the moderately and high-micronutrient deficient groups (Table 3; Figure 1A,B).

## 4. Discussion

This study, conducted on patients with stable HF followed in an outpatient clinic, showed a high prevalence of inadequate intake of vitamins B1, B2, B9, D, and E, and calcium, magnesium, and copper. We failed to confirm associations between inadequate micronutrient intake and the overall and event-free survival, when micronutrients were individually assessed by categories (adequate and inadequate) or evaluated by group of moderate or high inadequacies, especially those with a well-documented role in HF, for example, the B-complex vitamins, vitamin D, sodium, and zinc [13,15,31].

In line with our findings about the inadequate micronutrient intake, Lennie et al. [6] observed in patients with HF under outpatient follow up, who had also reduced LVEF, an inadequate intake of 21 micronutrients. A critical fact of our study was the high prevalence of inadequate intake of vitamin D, calcium, magnesium, copper, and low potassium, as recorded in a survey conducted in a similar population [9]. This finding requires attention given to the role of these micronutrients in cardiovascular health [15].

A possible explanation for the higher inadequate intake of these specific micronutrients in our study is the restriction placed on certain food sources, especially foods of animal origin, rich in saturated fatty acids—recommended as part of the HF treatment, mainly for ischemia patients with HF [32]. The high prevalence of inadequate intake of some B-complex vitamins observed in our study points to the low consumption of food sources, such as whole grains and offal observed in patients with HF [33]. Another factor is the recommendation of a low-sodium diet imposed on most patients with HF. In our study, the median daily intake was <2 g of sodium. A study of HF patients who were instructed to follow a 2 g sodium diet, certified that participants were unable to ingest more than 50% of the recommended vitamin D, vitamin E, magnesium and potassium. In addition, people on a low-sodium diet had lower intakes of calories, proteins, carbohydrates and fats, in addition to lower amounts of the micronutrients calcium, zinc and vitamin B1 [34].

Other reasons could explain this scenario of micronutrient intake inadequacies found in our study. In addition to the physiopathological alterations of HF [7,8], cultural and sociodemographic aspects may also interfere with food consumption, e.g., access to food in an adequate manner, both quantitatively and qualitatively. The Household Budget Survey, carried out in Brazil, showed that 36.7% of private households had some degree of food insecurity, with even greater proportions in the Northeast region, where hunger was present, with a severe Food Insecurity prevalence of 7.1% [35].

In our study, although we did not obtain statistical differences in the association between survival and nutrient intake, the percentage of event-free survival in the moderate deficiency group was higher than that in the high deficiency group. A previous study [6] evidenced that micronutrient deficiency was a predictor of event-free time in patients with HF, in which patients with a higher number of micronutrient deficiencies had lower event-free survival in the 1-year follow up. In addition, other authors found that dietary deficiency of water-soluble vitamins was adversely associated with the composite end point of all-cause mortality or hospitalization for cardiac- or HF-related reasons [36].

Therefore, these findings indicate the importance of an adequate intake of a set of micronutrients for the control of HF and the prevention of adverse events, such as hospitalization and mortality. These data also serve as a warning for the identification of patients with inadequate food consumption, who may be at greater risk of developing nutritional disorders, emphasizing the need for assessment and adequacy of nutritional guidelines for patients with HF. In our study, the large number of micronutrients with inadequate intake in the moderate (<14, minimum 9) and high (>14) deficiency groups, may explain the absence of associations between the groups of inadequate micronutrient intake and survival. Importantly, the median of 14 micronutrients with inadequate intake is higher than the value reported by Lennie et al. [6], whose median was 4 micronutrients with inadequate intake, and higher than the value evidenced by Lee et al., whose study found only 2 water-soluble vitamins with inadequate intake in the deficiency group.

Another factor that may explain the lack of association between micronutrient intake and clinical outcomes in our study is “metabolic flexibility.” This phenomenon is known as the capacity of the individual’s metabolism to adapt depending on the availability and needs of energy and nutrients for the detection of the substrate, traffic, storage, and utilization [37]. Preliminary studies with fat-soluble vitamins, for example, showed that there is interindividual variability in the bioavailability of vitamins A (β-carotene), D, and E and carotenoids (lutein and lycopene), which are modulated by single-nucleotide polymorphisms, which are close to genes that participate in intestinal uptake, efflux, metabolism, and transport, optimizing the bioavailability of these nutrients [38].

Additionally, although studies still focus on the importance of specific nutrients in the context of HF, it is increasingly evident that dietary patterns, more than a single nutrient, play a crucial role in the control of HF. The adoption of the Dietary Approach to Stop Hypertension and the Mediterranean diet has been emphasized as part of the treatment of patients with HF [39]. A prospective study including patients with HF treated in emergency departments during a mean follow up of 2 years found that adherence to the Mediterranean diet did not influence mortality after an episode of acute HF but was associated with a reduction in readmission during the following year [40]. Therefore, it reinforces the relevance of exploring dietary patterns in the context of HF for a better understanding of food and dietary intake in clinical outcomes.

Our study has some limitations, including the collection of outcome data from medical records, which can lead to incomplete gathering of some information, due to the lack of standardization or errors in typing, and the relatively short time of follow up, although some publications contemplate shorter follow-up time. The sample size and the absence of a group of individuals with adequate nutrient intake is also a limiting factor to assess the impact of micronutrient intake on overall and event-free survival. Another limitation is that the estimated average requirements used to establish adequacy of micronutrients intake were based on healthy populations [26,27,28], since there are no specific recommendations for individuals with HF.

To date, only few studies have evaluated the association between the prevalence of micronutrient inadequacy and clinical outcomes in patients with HF, especially in the northeast region of Brazil. Our study differs in the evaluation of the possible association between the total amount of deficient micronutrients in the diet and the adequacy of intake of each micronutrient individually treated with event-free time (hospitalization and mortality). This result greatly contributes to the knowledge of dietary intake in patients with HF and associations with clinical outcomes, with informative potential for professionals involved in the care of individuals with HF, in addition to guiding further research in this area.

## 5. Conclusions

Outpatients with HF have high prevalence of inadequate intake of various micronutrients. Inadequate micronutrient intake was not associated with hospitalization and mortality of outpatients with HF. Further studies, with higher numbers of HF patients, also including patients without micronutrients deficiencies, are needed to assess the impact of micronutrient deficiency and prognosis in this group of patients.

## Figures and Tables

**Table 1:** Sociodemographic, anthropometric, and clinical characteristics of patients with HF, distributed by micronutrient deficiency groups.

Variables	Overall (*n* = 121)	Moderate Deficiency (*n* = 67)	High Deficiency (*n* = 54)	*p*-Value
Sex ^a^				
Male	81 (66.9)	36 (44.4)	45 (55.6)	0.001
Female	40 (33.1)	31 (77.5)	9 (22.5)	
Age ^b^	55.8 (14.4)	55.09 (13.6)	56.8 (15.3)	0.51
Smoking ^a^				
Non-smoker	59 (59.4)	35 (59.3)	24 (40.7)	0.55
Ex-smoker	52 (44.4)	29 (55.8)	23 (44.2)	
Smoker	6 (5.1)	2 (33.3)	4 9 (66.7)	
Drinking ^a^				
Ex-drinker	64 (55.2)	31 (48.4)	33 (51.6)	0.04
Has never drunk/does not drink	52 (44.8)	35 (67.3)	17 (32.7)	
BMI (kg/m^2^) ^b^		26.97 (5.12)	26.71 (5.1)	0.79
BMI classification ^a^				
Underweight	12 (10.5)	5 (41.7)	7 (58.3)	0.52
Normal weight	47 (41.2)	28 (59.6)	19 (40.4)	
Overweight/obese	55 (48.2)	32(58.2)	23 (41.8)	
Etiology ^a^				
Ischemic	49 (40.5)	33 (67.3)	16 (32.7)	0.003
Nonischemic	56 (46.3)	31 (55.4)	25 (44.6)	
Diagnosis of LVEF ^a^				
HFrEF	67 (59.3)	35 (52.2)	32 (47.8)	0.11
HFmEF	21 (18.6)	16 (76.2)	5 (23.8)	
HFpEF	25 (22.1)	12 (48.0)	13 (52.0)	
NYHA functional class ^a^				
I/II	101 (88.6)	56 (55.4)	45 (44.6)	0.68
III/IV	13 (11.4)	8 (61.5)	5 (38.5)	
Comorbidities ^a^				
Arterial hypertension				
No	40 (33.9)	21 (52.5)	19 (47.5)	0.59
Yes	78 (66.1)	45 (57.7)	33 (42.3)	
Diabetes mellitus				
No	84 (71.2)	41 (48.8)	43 (51.2)	0.01
Yes	34 (28.8)	25 (73.5)	9 (26.5)	
eGFR < 60 mL/min/1.73 m^2^				
No	66 (60.6)	39 (59.1)	27 (40.9)	0.92
Yes	43 (39.4)	25 (58.1)	18 (41.9)	
Medication ^a^				
ARB/ACEI	104 (89.7)	58 (55.8)	46 (44.2)	0.87
Diuretics	94 (81)	52 (55.3)	42 (44.7)	0.75
Beta-blockers	111 (94.9)	63 (56.8)	48 (43.2)	0.40
Hypoglycemics	26 (23.9)	20 (76.9)	6 (23.1)	0.02
Hypolipidemics	61 (56.0)	36 (59.0)	25 (41.0)	0.61
Antiplatelet drugs	51 (44.0)	33 (64.7)	18 (35.3)	0.10
Clinical outcomes ^a^				
No events	96 (79.3)	52 (43.0)	44 (36.4)	0.69
Hospitalization	8 (6.6)	4 (3.3)	4 (3.3)	
Death	17 (14.0)	7 (5.8)	10 (8.3)	

^a^ Data presented as *n* (%); ^b^ data presented as mean (standard deviation) with a *p*-value based on the t-test. Missing data for variables: BMI (*n* = 7), alcoholism (*n* = 5), NYHA functional class (*n* = 7), diagnosis of LVEF (*n* = 8), presence of arterial hypertension and diabetes (*n* = 3), eGFR < 60 mL/min/1.73 m^2^ (*n* = 12), medications (*n* = 12), and undefined etiology (*n* = 16). BMI, body mass index; LVEF, left ventricular ejection fraction; HFpEF, heart failure with preserved ejection fraction; HFmEF, heart failure with mid-range ejection fraction; HFrEF, heart failure with reduced ejection fraction; NYHA, New York Heart Association; eGFR, estimated glomerular filtration rate; ARB/IECA, angiotensin receptor blocker II/angiotensin-converting enzyme inhibitor. Moderate deficiency group: there was inadequate intake of up to 14 nutrients; high deficiency group: there was inadequate intake of more than 14 nutrients.

**Table 2:** Daily nutritional recommendations, micronutrient intake, and prevalence of inadequate micronutrient intake (% IN), by sex and age group, in patients with HF.

Micronutrients *	Male						Female					
	EAR ^a^	Mean (SD ^b^)	P10 ^c^	P50	P90	%IN ^d^	EAR	Mean (SD)	P10	P50	P90	%IN
Vitamin A (mcg ^e^)	625	823.1 (624.4)	336.4	665.5	1601.6	37.5	500	690.9 (274.0)	355.3	699.2	1046.7	24.2
Vitamin B1 (mg ^f^)	1	0.2 (0.2)	0.0	0.2	0.5	100	0.9	0.2 (0.2)	(−0.0)	0.2	0.5	100
Vitamin B2 (mg)	1.1	0.4 (0.3)	0.1	0.4	0.8	98.5	0.9	0.4 (0.3)	0.0	0.4	0.7	98.42
Vitamin B3 (mg)	12	15.0 (6.2)	7.3	14.8	355.4	31.2	11	16.0 (4.9)	10.5	15.055	22.8	17.1
Vitamin B6 (mg)												
19 to 50 years old	1.1	1.7 (0.5)	1.2	1.7	2.6	11.3	1.1	1.6 (0.3)	1.2	1.6	2.0	5.1
>51 years old	1.4	1.7 (0.5)	1.2	1.7	2.3	24.8	1.3	1.8 (0.5)	1.3	1.8	2.3	13.79
Vitamin B9 (mcg)	320	124.5 (63.6)	64.3	112.0	215.6	99.9	320	117.0 (58.5)	53.1	100.905	205.2	100.0
Vitamin B12 (mcg)	2	3.3 (2.2)	1.5	2.7	4.8	28.4	2	0.4 (0.3)	0.0	0.4	0.7	21.5
Vitamin C (mg)	75	187.7 (164.6)	45.1	139.9	391.0	24.8	60	135.9 (82.1)	37.6	127.2	242.5	17.6
Vitamin D (mcg)	10	1.9 (1.2)	0.4	1.7	3.7	100	10	2.1 (1.7)	0.2	1.8	4.9	100
Vitamin E (mg)	12	6.8 (3.7)	3.4	6.3	9.972	91.9	12	7.6 (3.5)	4.8	6.7	10.6	89.1
Calcium (mg)												
19 to 50 years old	800	368.9 (123.7)	207.9	368.2	582.9	99.7	800	363.3 (106.7)	239.1	365.9	542.3	100.0
>51 years old	1000	391.6 (166.3)	204.8	353.6	593.9	100.0	1000	473.9 (168.2)	222.4	510.6	698.7	99.9
Copper (mg)	0.7	0.2 (0.3)	(−0.0)	0.2	0.6	96.3	0.7	0.2 (0.2)	(−0.0)	0.2	0.5	99.2
Iron (mg)												
19 to 50 years old	6	35.9 (169.9)	(−22.8)	6.8	17.7	38.6	8.1	18.5 (18.0)	(−1.3)	22.5	37.4	2.7
>51 years old	6	23.6 (104.7)	(−9.5)	10.8	28.9		5	23.1 (31.2)	(−6.4)	19.1	33.4	6.3
Phosphorus (mg)	580	814.3 (185.9)	605.4	796.7	1058.0	10.4	580	808.4 (144.6)	633.7	790.1	1054.5	5.7
Iodine (mcg)	95	162.6 (95.8)	49.2	146.3	307.0	23.9	95	156.9 (59.6)	86.5	145.205	229.2	44.4
Magnesium (mg)												
19 to 30 years old **	330	197.4 (50.5)	146.3	244.7	198.7	99.6	265	188.9 (42.5)	136.8	180.0	242.0	96.3
>31 years old	350	188.6 (47.6)	145.3	183.2	248.5	100.0						
Selenium (mcg)	45	55.3 (20.6)	32.2	50.5	80.2	30.9	45	53.6 (18.4)	33.5	51.4	83.1	31.9
Sodium (mg) ***	2800	1784.9 (658.2)	924.7	1748	2501.2	3.7	2800	1758.7 (420.2)	1089.0	1776.2	2254.3	0.0
Zinc (mg)	9.4	7.3 (2.3)	4.8	7.1	10.3	82.4	6.8	7.0 (1.7)	5.3	6.8	9.6	44.0

^a^ EAR, estimated average requirement; ^b^ SD, standard deviation; ^c^ P, percentile; ^d^ IN, inadequate; ^e^ mcg, micrograms; ^f^ mg, miligrams. * Vitamins A, B1, B2, B3, B9, B12, C, D, and E and elements copper, phosphorus, iodine, selenium, and zinc: recommendations for the age group from 19 to 71 years old. ** It was not possible to calculate the prevalence of inadequacy of magnesium for females aged 19 to 30 years. *** Sodium recommendation according to the according to the Brazilian HF guidelines [1].

**Table 3:** Overall and event-free survival within 24 months in patients with HF, considering nutrient adequacy and micronutrient deficiency groups.

Variables	Overall Survival			Event-Free Survival		
	% Survival (95% CI)	HR (95% CI)	*p*	% Survival (95% CI)	HR (95% CI)	*p*-Value
Vitamin A						
Adequate	80.08 (66.74–88.51)	1.00	0.94	77.62 (64.39–86.44)	1.00	0.18
Inadequate	81.80 (63.22–91.57)	1.04 (0.38–2.81)		67.24 (49.04–80.16)	1.69 (0.77–3.71)	
Vitamin B2						
Adequate	100 (0)	#	#	100 (0)	#	#
Inadequate	80.00 (69.60–87.16)	#		72.96 (62.45–80.96)	#	
Vitamin B3						
Adequate	78.99 (66.97–87.05)	1.33 (0.38–4.64)	0.65	73.44 (61.40–82.25)	1.00	0.93
Inadequate	87.00 (63.07–95.88)	1.00		74.94 (51.21–88.31)	1.04 (0.41–2.61)	
Vitamin B6						
Adequate	80.48 (69.61–87.80)	1.00	0.75	74.49 (63.47–82.63)	1.00	0.34
Inadequate	88.82 (62.07–97.09)	1.26 (0.28–5.60)		76.63 (48.60–90.64)	1.68 (0.57–4.94)	
Vitamin B9						
Adequate	100 (0)	#	#	100 (0)	#	#
Inadequate	80.33 (70.06–87.38)	#		73.43 (63.04–81.32)	#	
Vitamin B12						
Adequate	78.18 (66.27–86.31)	1.78 (0.41–7.81)	0.43	72.33 (60.32–81.26)	1.00	0.94
Inadequate	91.83 (71.08–97.89)	1.00		80.77 (59.81–91.51)	1.03 (0.38–2.76)	
Vitamin C						
Adequate	82.09 (71.48–89.05)	1.00	0.36	75.20 (64.36–83.17)	1.00	0.42
Inadequate	66.96 (26.05–88.73)	1.78 (0.51–6.24)		60.88 (24.29–84.02)	1.54 (0.53–4.52)	
Vitamin E						
Adequate	53.33 (6.83–86.31)	2.94 (0.67–12.92)	0.13	53.33 (6.83–86.31)	1.92 (0.45–8.18)	0.37
Inadequate	81.96 (71.65–88.80)	1.00		74.70 (64.20–82.52)	1.00	
Calcium						
Adequate	100 (0)	#	#	100 (0)	#	#
Inadequate	80.31 (70.03–87.37)	#		73.40 (63.00–81.29)	#	
Iron						
Adequate	76.32 (63.01–85.38)	2.51 (0.72–8.76)	0.13	68.88 (55.70–78.86)	1.00	0.12
Inadequate	89.29 (70.08–96.46)	1.00		83.70 (64.96–92.93)	2.11 (0.79–5.65)	
Phosphorus						
Adequate	79.87 (69.44–87.07)	#	#	72.78 (62.24–80.82)	#	#
Inadequate	100 (0)	#		100 (0)	#	
Iodine						
Adequate	82.21 (71.18–89.32)	1.00	0.41	74.70 (63.42–82.96)	1.00	0.62
Inadequate	72.27 (39.55–89.25)	1.60 (0.52–4.93)		68.99 (38.28–86.63)	1.27 (0.47–3.41)	
Magnesium						
Adequate	50.00 (0.01–0.91)	3.23 (0.42–24.50)	0.23	50.00 (0.60–91.04)	2.06 (0.27–15.28)	0.47
Inadequate	81.30 (71.08–88.21)	1.00		74.25 (63.86–82.06)	1.00	
Manganese						
Adequate	77.43 (53.98–89.93)	1.06 (0.37–3.01)	0.91	67.51 (45.25–82.31)	1.25 (0.54–2.90)	0.60
Inadequate	82.10 (70.29–89.55)	1.00		76.32 (64.39–84.71)	1.00	
Potassium						
Adequate	0 (0)	8.82 (1.08–72.14)	0.01 *	0.00	5.00 (0.65–38.38)	0.09
Inadequate	81.51 (71.39–88.34)	1.00		86.58 (78.30–91.86)	1.00	
Selenium						
Adequate	76.78 (63.76–85.63)	2.05 (0.59–7.15)	0.25	69.20 (56.19–79.05)	1.80 (0.68–4.82)	0.23
Inadequate	89.94 (71.00–96.77)	1.00		84.27 (65.72–93.26)	1.00	
Sodium						
Adequate	80.25 (69.96–87.33)	#	#	73.30 (62.89–81.22)	#	#
Inadequate	100.00 (0)	#		100 (0)	#	
Zinc						
Adequate	78.25 (57.60–89.67)	1.28 (0.47–3.49)	0.62	75.81 (55.57–87.76)	1.00	0.89
Inadequate	81.48 (68.74–89.41)	1.00		72.70 (59.91–82.00)	1.06 (0.44–2.55)	
Micronutrient deficiency groups						
Moderate	79.71 (61.93–89.82)	1.00	0.91	79.71 (61.93–89.82)	1.00	0.26
High	81.30 (67.50–89.66)	0.94 (0.36–2.48)		74.38 (61.22–83.65)	1.63 (0.68–3.92)	

CI, confidence interval; HR, hazard ratio. * Significant difference using the log-rank test; # unable to calculate (no events were observed in one of the categories). Calculating the HR and log-rank for vitamins B1, B5, and D and copper was not possible (absence of patients in the appropriate category). Event-free survival: survival time without adverse effects (hospitalization and mortality). Moderate deficiency group: inadequate intake of up to 14 nutrients; High deficiency group: inadequate intake of more than 14 nutrients.

## Data Availability

The data presented in this study are available on request from the corresponding author. The data are not publicly available due to privacy restrictions.
